# Recent advances in alcoholic hepatitis

**DOI:** 10.12688/f1000research.20394.1

**Published:** 2020-02-10

**Authors:** Vikrant Rachakonda, Ramon Bataller, Andres Duarte-Rojo

**Affiliations:** 1 Division of Gastroenterology and Hepatology, Starzl Transplantation Institute, and Pittsburgh Liver Research Center, University of Pittsburgh, Pittsburgh, PA, 15213, USA

**Keywords:** addiction, corticosteroids, biomarkers, liver transplantation

## Abstract

Alcoholic hepatitis is the severest clinical presentation of alcoholic liver disease. Lacking an effective pharmacologic treatment, alcoholic hepatitis is associated with a poor prognosis and its recovery relies mostly on abstinence. With alcohol use disorder being universally on the rise, the impact of alcoholic hepatitis on society and health-care costs is expected to increase significantly. Prognostic factors and liver biopsy can help with timely diagnosis, to determine eligibility and response to corticosteroids, and for prognostication and transplant referral. Although recent discoveries in the pathophysiology of alcoholic hepatitis are encouraging and could pave the way for novel treatment modalities, a multidisciplinary approach considering timely identification and treatment of liver-related complications, infectious and metabolic disease, malnutrition, and addiction counseling should be emphasized. Apart from proper selection of candidates, transplant programs should provide adequate post-transplant addiction support in order to make of early liver transplantation for alcoholic hepatitis the ultimate sobering experience in the next decade.

## Introduction

Alcoholic hepatitis (AH) is a life-threatening form of acute-on-chronic liver failure that occurs in patients with sustained heavy alcohol use. AH is characterized by a rapid onset or worsening of jaundice, liver synthetic dysfunction, and features of hepatic decompensation, including ascites, encephalopathy, and portal hypertensive bleeding. An episode of AH is frequently the first clinical presentation of alcohol-related liver disease, and in patients with severe disease, prognosis is poor; mortality is 20 to 50% at 28 days and up to 70% at 90 days ^1, 2^. Pharmacotherapy for AH remains limited to corticosteroids; however, almost half of patients cannot tolerate or do not respond to corticosteroid therapy. The only intervention shown to improve long-term survival in AH is alcohol abstinence, thus highlighting a critical role for multidisciplinary care incorporating mental health and addiction teams. More recently, early liver transplantation (LT) has been used in carefully selected populations showing 3-year survival rates equivalent to those for other chronic liver diseases; concerns regarding post-transplant alcohol relapse, however, have limited acceptance of early transplantation as standard treatment.

## Epidemiology of alcoholic hepatitis

The epidemiology of AH is poorly understood, particularly in the US, where liver biopsy is rarely performed for this condition. Trends in patterns of alcohol use, however, suggest that AH incidence rates are likely to increase in Western nations. Recent US reports on the impact of state alcohol sales policies have highlighted increased rates of alcohol consumption ^3, 4^. Furthermore, drinking at an earlier age ^5, 6^ and binging patterns of use have dramatically increased in the last decade ^7^. These behaviors have translated into increased mortality related to alcoholic liver disease; from 2009 to 2016, Americans 25 to 34 years old experienced the greatest increase in liver-related mortality among all age groups and this increase was driven entirely by alcoholic liver disease ^8^. Although a significant proportion of these deaths are likely attributable to AH, no US studies have systematically explored the epidemiology of AH. In a Danish national cohort study, the incidence of AH rose from 24 to 34 per million women and from 37 to 46 per million men between 1999 and 2008 ^9^, and it is likely that similar trends have occurred in other Western nations.

With the changing epidemiology of alcohol use and alcohol-related liver disease, the health-care and economic burden of AH has increased over the last decade. For example, an analysis of the Nationwide Inpatient Sample demonstrated increased rates of hospitalization for AH from 2002 to 2010 ^10^. While there was a significant decline in inpatient mortality due to AH (4.3% absolute risk reduction), the cost of hospitalization increased by 41% ^10^. Another report showed a 96% increase in the inpatient cost of care for alcoholic cirrhosis between 2002 and 2014, representing almost 50% of the total inpatient charges for cirrhosis ^11^. As length of stay remained unchanged over the studied time frame for both studies, increased costs likely represent an absolute increase in resource utilization.

## Pathophysiology of alcoholic hepatitis

AH is driven by both intra- and extra-hepatic effects of alcohol. Within hepatocytes, alcohol is degraded into the toxic metabolite acetaldehyde via non-inducible (alcohol dehydrogenase) and inducible (CYP2E1) enzymes. In addition to acetaldehyde, multiple reactive oxygen intermediates (ROIs) are generated by CYP2E1. Acetaldehyde directly induces hepatocyte death through both necrosis and apoptosis, and hepatocyte injury leads to release of damage-associated molecular patterns (DAMPs). DAMPs subsequently bind to Toll-like receptors (TLRs) in Kupffer cells, the resident tissue macrophage of the liver, leading to activation of the inflammasome, a multiprotein complex containing caspase 1. Upon inflammasome activation, caspase 1 generates the pro-inflammatory cytokine interleukin 1 beta (IL-1β) ^12– 14^.

Alcohol exerts deleterious intestinal effects, which eventually lead to hepatic inflammation through multiple mechanisms. Particularly, alcohol exerts direct toxicity on intestinal epithelial cells and decreases the expression of tight-junction proteins, which enhances the permeability of the intestinal mucosa, permitting translocation of pathogen-associated molecular patterns (PAMPs) such as lipopolysaccharide (LPS) into the liver ^15– 17^. As with DAMPs, PAMPs can then activate macrophage inflammasomes via TLR, leading to elaboration of multiple inflammatory cytokines (including IL-1B, tumor necrosis factor alpha (TNF-α) and IL-10) and neutrophil chemokines (including IL-8, Gro-α, CXCL-5, and CXCL-6) ^18^. The net increase in intra-hepatic inflammation perpetuates further hepatocellular mitochondrial dysfunction, ROI generation, and hepatic failure.

In addition to dysregulated hepatic inflammation, multiple clinical observations point to hepatocellular dysfunction and impaired regeneration as critical drivers of liver failure in AH. First, liver explants from AH patients undergoing LT demonstrate near complete loss of hepatocyte proliferation compared with alcoholic cirrhosis and normal liver ^19, 20^. In addition, hepatocyte proliferation is associated with improved survival in severe AH ^20^. Finally, patients with AH exhibit a significant expansion of liver progenitor cells (LPCs) (termed ductular reaction) and failure of LPC differentiation into mature hepatocytes correlates with reduced survival ^21^. To further delineate molecular mechanisms of hepatocellular dysfunction in AH, Argemi *et al*. performed untargeted RNA sequencing in livers with different alcoholic liver disease phenotypes and identified that development of AH was characterized by defective activity of liver-enriched transcription factors ^22^. In particular, transforming growth factor beta 1 (TGF-β1) was a primary transcriptional regulator in AH and induced fetal isoforms of hepatic nuclear factor 4a (HNF-4a) P2 promoter, resulting in defective hepatic synthetic and metabolic function. Altered expression of HNF-4a–dependent genes was driven by epigenetic phenomena ^22^. Together, these findings highlight a critical role for not only intra-hepatic inflammation but also hepatocellular dysfunction and impaired regeneration in the pathogenesis of AH.

Recently, genetic predisposition has expanded our understanding of alcoholic liver disease, and some relevant genetic signals are shared with the non-alcoholic steatohepatitis counterpart (for example, patatin-like phospholipase domain-containing protein 3, transmembrane 6 superfamily member 2, and hydroxysteroid 17-beta dehydrogenase 13) ^23– 25^. Although the impact of genetic predisposition in AH is less clear, mutations in CYP2E1 ^26^ and, more recently, a transcriptome analysis were over-represented in AH or had prognostic implications ^27^. In the latter, a 123-gene prognostic signature was identified from fixed liver biopsy samples in patients with severe AH and it improved the prediction of transplant-free survival along with the model for end-stage liver disease (MELD). The identified genetic signals corresponded to inflammatory and oxidative stress pathways, intermediate metabolism, and hepatic stellate cells ^27^.

Gut dysbiosis has been involved in the pathophysiology of various chronic liver ailments, including alcoholic liver disease ^28^. Alcohol directly contributes to intestinal microbial dysbiosis, and in both murine and human studies, alcohol induces shifts in bacterial communities favoring pathogenic species ^29, 30^. Non-bacterial populations are also affected, as a recent translational study described reduced fungal biodiversity with enhanced anti-fungal immune responses in severe AH ^31^. Such changes in microbiota break the balance of the gut–liver axis either directly through bacterial metabolites or indirectly through TLRs and other signaling molecules (PAMP/DAMP pathways) and activation of the immune system ^32^. The beneficial effects that modulation of the intestinal flora brings to patients with AH, as observed in pilot trials using either rifaximin or fecal microbiota transplantation, add evidence to support the pivotal role of the gut–liver axis and gut eubiosis as a novel therapeutic aim ^33, 34^.

## Diagnostic and prognostic assessment in alcoholic hepatitis

There are no reliable non-invasive markers for identification of AH and hence the syndrome is most often diagnosed on clinical grounds. The cardinal feature of AH is recent onset of jaundice within 60 days of last alcohol consumption in a patient with known heavy alcohol use for more than 6 months. The diagnosis relies on proving heavy alcohol use as the primary etiology of liver injury while excluding other causes of liver disease. Although minimum alcohol use requirements for AH have not been described, a consumption threshold of at least three drinks (40 g) daily in women or at least four drinks (60 g) daily in men is most commonly used for inclusion in treatment studies in AH. Laboratory features of AH include serum bilirubin of at least 3 mg/dL, aspartate aminotransferase (AST) between 50 and 400 IU/mL, and AST/alanine aminotransferase ratio of at least 1.5.

In 2016, an expert panel within the National Institute for Alcohol Abuse and Alcoholism proposed a classification scheme for AH as follows: (1) definite AH: clinical criteria for diagnosis are met and confirmed by liver biopsy; (2) probable AH: clinically diagnosed AH without liver biopsy and without confounding factors; and (3) possible AH: clinically diagnosed AH but with confounding factors, including possible ischemic hepatitis, drug-induced liver injury, atypical laboratory tests, or uncertainty regarding alcohol consumption or a combination of these ^2^; in this subset, liver biopsy is recommended.

In contrast, the European Association for the Study of the Liver recommends liver biopsy for the diagnosis of AH, as the accuracy of clinical diagnosis is around 70 to 80% ^35^. Typical histologic findings include macrovesicular steatosis, ballooning degeneration of hepatocytes, Mallory–Denk bodies, megamitochondria, lobular inflammation with neutrophilic satellitosis, and bilirubinostasis with ductular reaction ^35– 37^. Advanced fibrosis is a common feature of AH, and it is estimated that 80% of patients with AH have micronodular cirrhosis ^37^. Typical fibrosis patterns include centrilobular, pericellular, and sinusoidal fibrosis (“chicken wire” fibrosis). A landmark study from 2014 demonstrated that, in addition to prognostic information, histology may also provide prognostic value in AH, and a scoring system consisting of fibrosis, bilirubinostasis, neutrophilic infiltration, and megamitochondria demonstrated an area under the receiver operating characteristic curve (AUROC) of 0.77 for 90-day mortality ^37^. Severe AH was defined as a histologic score of 6 to 9 and was associated with 50% 90-day mortality ^37^. Interestingly, severity of fibrosis was the strongest negative predictor of survival whereas neutrophilic inflammation was associated with improved survival. Despite the utility of liver biopsy, the presence of coagulopathy, thrombocytopenia, uremia, and ascites in AH generally precludes percutaneous liver biopsy, and transjugular biopsy is not widely available.

Several laboratory-based scoring systems have been studied for outcome prediction in AH and these are highlighted in Table 1. Historically, the Maddrey discriminant function (MDF) was the most commonly used model to identify candidates for corticosteroid therapy ^38^. Two major drawbacks of MDF were (1) the use of prothrombin time, which is not standardized between clinical laboratories, and (2) the absence of renal function assessment, as acute kidney injury is a strong predictor of mortality in AH ^39^. More recently, the MELD score has been used to classify AH patients at high risk of death in need of treatment ^40^. At a cutoff of at least 21, MELD score exhibited 75% sensitivity and specificity for predicting 90-day mortality. Other models, including the age/bilirubin/international normalized ratio/creatinine (ABIC) score ^41^ and the Glasgow Alcoholic Hepatitis Score (GAHS) ^42^ are less well studied in North American populations. An ABIC score of less than 6.71 has been associated with 100% survival, whereas an ABIC score of more than 9.00 predicts 25% survival at 90 days ^41^. The GAHS has also been used to identify candidates for corticosteroid therapy, as patients with a GAHS of at least 9 and an MDF of at least 32 exhibited better survival with steroid therapy than without, whereas subjects with a GAHS of less than 9 and an MDF of less than 32 did not benefit from treatment ^43^.

**Table 1.** Prognostic scoring systems for alcoholic hepatitis.

Score	Formula				Interpretation
MDF	4.6×(PT-control PT)+bilirubin (mg/dL)				Severe: ≥32
MELD	3.8×[ln(bilirubin(mg/dL)]+11.2×[ln(INR)]+9.6×[ln(creatinine(mg/dL)]+6.4				Severe: ≥21
ABIC	(age×0.1)+(bilirubin×0.08)				Low risk: ≤6.71 Severe: >9.0
GAHS		1	2	3	Severe: ≥9
	Age	<50	≥50	–	
	WBC count	<15	≥15	–	
	Urea, mmol/L	<5	≥5	–	
	INR	<1.5	1.5–2.0	>2.0	
	Bilirubin, mg/dL	<7.3	7.4–14.6	>14.6	

ABIC, age/bilirubin/international normalized ratio/creatinine; GAHS, Glasgow Alcoholic Hepatitis Score; INR, international normalized ratio; MDF, Maddrey discriminant function; MELD, Model for End-Stage Liver Disease; PT, prothrombin time; WBC, white blood cell.

Early observational trials of steroid therapy identified declining bilirubin as a marker of therapeutic response and hence the Lille score was developed to quantify changes in labs over 7 days of steroid treatment. A Lille score of more than 0.45 after 7 days was associated with over 75% 90-day mortality ^44^. A multinational study reported on a similar accuracy when the Lille score was evaluated at day 4, when compared with the original one after 7 days on corticosteroids ^45^. Combining MELD and Lille scores increases prognostic accuracy compared with either score alone ^46^, and various useful combination strategies were recently studied as part of a *post hoc* analysis of a large multicenter AH clinical trial ^47^.

Given the above limitations of laboratory-based models for outcome prediction in AH, a number of studies have explored novel biomarkers of AH. For example, analysis of circulating exosomes has demonstrated increased enrichment of miR-155 (a key regulator of inflammation), miR-192, and miR-30a in patients with AH compared with controls ^48, 49^. Circulating levels of several cytokines, including TNF-α, IL-1, IL-6, and IL-8, have also been studied in AH, and elevated serum IL-6 levels, in particular, have shown promise as a marker of increased mortality in patients with an MDF of at least 32 ^50^. More recently, two studies have shown that increased levels of cytokeratin 18 fragments may be used for identification and severity assessment in AH ^51, 52^, and urinary metabolomics studies have identified higher levels of urinary acrolein metabolites in severe AH compared with both healthy controls and non-severe disease ^53^. Finally, an analysis of blood neutrophil/lymphocyte ratio (NLR) demonstrated that NLR was associated with infection, acute kidney injury, and steroid responsiveness ^54^. Validation and standardization of these promising tools are still needed across multiple clinical settings.

## Management of acute alcoholic hepatitis

The pharmacotherapy for AH has changed little since the introduction of corticosteroids in the early 1970s ^55^, and although an early clinical trial demonstrated that pentoxifylline decreased short-term mortality by reducing rates of renal failure ^56^, the recently reported STOPAH (steroids or pentoxifylline for alcoholic hepatitis) study has raised concerns regarding the efficacy of both treatments ^57^. Over 1,100 patients were enrolled from hospitals throughout Britain and randomly assigned into a 2×2 factorial designed clinical trial to study the benefit of corticosteroids and pentoxifylline. Corticosteroids demonstrated a trend toward reduced 28-day mortality compared with placebo (14% versus 18%, *P* = 0.06), but no reduction was observed for 90-day mortality. Pentoxifylline provided no improvements in either short-term or long-term mortality. Importantly, mortality rates were significantly lower than those reported in other clinical trials of severe AH. As biopsy-proven AH was not used as an inclusion criterion, this raised concerns regarding whether a subset of patients had acute-on-chronic liver failure related to decompensated cirrhosis as opposed to AH. Two recent meta-analyses of randomized controlled trials reported that corticosteroids provided significant survival benefit at 28 days but not at 90 or 180 days ^58, 59^, although the last updated Cochrane meta-analysis continues to find no benefit. Given the short-term benefits of corticosteroids, their judicious use is still warranted in selected populations. ****

There is increasing interest in novel treatments for severe AH ( Table 2). Ongoing trials are exploring the efficacy of antibiotic therapy, farsenoid X receptor (FXR) agonists (obeticholic acid), caspase inhibitors (emricasan), receptor tyrosine kinase inhibitors (selonsertib), IL-1R antagonists (anakinra), and IL-22 agonists. Of particular interest is the use granulocyte-colony stimulating factor (G-CSF). The rationale for G-CSF is based on (1) histologic studies demonstrating that increased hepatic neutrophil infiltration is associated with improved survival in AH ^37^ and (2) pre-clinical observations that G-CSF mobilizes granulocytes to improve survival in acute-on-chronic liver failure ^60^. A few randomized pilot studies from India have shown increased survival and more rapid decline in MELD scores in both decompensated cirrhosis and AH ^61– 65^. Though promising, these results will require replication in other clinical centers.

**Table 2.** Recent/ongoing clinical trials for treatment of alcoholic hepatitis.

Agent	Mechanism of action	Study design	Inclusion criteria	Primary endpoint	ClinicalTrials.gov identifier and status
G-CSF	Hepatic neutrophil expansion, liver regeneration	Placebo-controlled RCT: + CS in PR No CS in NR	DF ≥32	NR: 2-month survival PR: 6-month survival	NCT02442180, phase IV, enrolling
OCA	FXR agonist	Placebo-controlled RCT	MELD 11–20	MELD change after 6 weeks	NCT02039219, phase II, completed
Amoxicillin/ Clavulanic acid	Gut microbiome modulation, infection prevention	Placebo-controlled RCT with CS	MELD ≥21	2-month survival	NCT02281929, phase III, active, not enrolling
Emricasan	Caspase inhibitor	Placebo-controlled RCT	MELD 20–35 or MELD >35 plus SOFA <10	28-day survival	NCT01912404, phase II, terminated
Selonsertib	ASK-1 inhibitor, MAPK inhibitor	Placebo-controlled RCT with CS	DF ≥32	Safety at 28 days	NCT02854631, phase II, completed
Anakinra	IL-1R antagonist	RCT of Anakinra + Zinc + PTX versus CS alone	MELD ≥20 and DF ≥32	6-month survival	NCT01809132, phase II, completed
IL-22	Hepatic regeneration	Open-label	MELD 11–28	Safety at 42 days	NCT unavailable, phase I completed
FMT	Alleviates gut dysbiosis	Open-label	DF ≥32 and low- grade HE	3-month survival	NCT03827772, phase II, enrolling

ASK-1, apoptosis signal-regulating kinase-1; CS, corticosteroids; DF, discriminant function; FMT, fecal microbiota transplantation; FXR, farsenoid X receptor; G-CSF, granulocyte colony-stimulating factor; HE, hepatic encephalopathy; IL-1R, interleukin-1 receptor; IL-22, interleukin-22; MELD, Model for End-Stage Liver Disease; NR, non-responder; OCA, obeticholic acid; PR, partial responder; PTX, pentoxifylline; RCT, randomized controlled trial; SOFA, sequential organ failure assessment.

## Supportive measures

### Alcohol abstinence

Although corticosteroids marginally improve short-term mortality, only alcohol abstinence has been shown to increase 6-month survival ^66, 67^. Most patients with AH meet criteria for an alcohol use disorder (AUD), characterized by ongoing alcohol consumption despite experiencing adverse consequences of drinking ^68^. Therefore, use of screening tools to diagnose AUDs in patients with AH is critical to guide referral to optimal treatment pathways. One recent meta-analysis of the AUD Identification Test-Consumption (AUDIT-C) ^69^ ( https://www.drugabuse.gov/sites/default/files/files/AUDIT.pdf) demonstrated feasibility and efficacy of this instrument for identification of AUDs; furthermore, counseling interventions in those patients testing positive led to reductions in harmful alcohol consumption ^70^.

In addition to addiction counseling, pharmacotherapy for AUD is now available ( Table 3). Although disulfram and naltrexone have US Food and Drug Administration approval for treatment of AUD, both drugs exhibit significant hepatotoxicity limiting their use in advanced liver disease. Baclofen has been studied extensively for AUD treatment in patients. Given the limited hepatic metabolism of baclofen, there is a theoretically lower risk of hepatotoxocity. Results of baclofen treatment trials, however, are mixed ^71^. Whereas one meta-analysis of 14 studies demonstrated no superiority of baclofen over placebo ^72^, two other meta-analyses demonstrated increased rates of abstinence and increased time to first relapse with baclofen ^73, 74^. Other drugs with low reported risk of hepatic injury include acamprosate, gabapentin, topiramate, and varenicline. However, of these agents, only acamprosate is approved for treatment of AUD. A recent meta-analysis demonstrated reduced rates of alcohol relapse for acamprosate (number needed-to-treat of 12; 27 trials) ^75^. Although further trials are needed to assess efficacy specifically in alcoholic liver disease, acamprosate represents an alternative therapeutic option in survivors of an episode of severe AH.

**Table 3.** Pharmacotherapy for alcohol use disorder.

Drug	Mechanism of action	Dose	Hepatic metabolism and risk of toxicity
FDA-approved			
Acamprosate	NDMA agonist	666 mg orally three times daily (333 mg with renal dysfunction)	No hepatic metabolism, adjust dose in kidney injury
Disulfram	Acetaldehyde dehydrogenase inhibitor	250–500 mg orally daily	No hepatic metabolism, associated with drug- induced liver injury
Naltrexone	Mu-opiate receptor antagonist	50 mg orally daily or 380 mg intra-muscularly monthly	Hepatocellular injury described
Not FDA-approved			
Baclofen	GABA-B receptor agonist	Up to 80 mg daily orally in divided doses	Minimal hepatic metabolism; formally studied in cirrhosis
Gabapentin	GABA modulator	900–1800 mg daily in divided doses	No hepatic metabolism; sedation risk in cirrhotic patients with hepatic encephalopathy
Topiramate	Anticonvulsant	300 mg orally daily	Partial hepatic metabolism via glucoronidation
Varenicline	Nicotinic receptor agonist	2 mg orally daily	Minimal hepatic metabolism

FDA, US Food and Drug Administration; GABA, gamma aminobutyric acid; NMDA, *N*-methyl- d-aspartate.

### Identification and treatment of infections

Infections are a common complication of AH and contribute to increased mortality in AH ^76^. In the STOPAH trial, infections were responsible for 24% of all deaths ^57^. Patients with severe AH are at particularly increased risk for infection for a number of reasons. First, immune function of circulating innate immune cells is impaired, as multiple studies have identified decreased phagocytic activity, oxidative burst, and proliferation in severe AH ^77, 78^. Second, immunosuppression with corticosteroids impacts infection risk, as shown in a subanalysis of the STOPAH trial where circulating bacterial DNA detected at enrollment was associated with development of early infections (within 7 days of commencing treatment) only among patients who received prednisolone ^79^. The source and type of infection also change during the disease course. In an early study of AH, spontaneous bacterial peritonitis and spontaneous bacteremia were leading infections at initial presentation whereas respiratory infections were the most common infections after exposure to corticosteroids ^80^. Although Gram-negative bacteria are the leading organisms identified in AH, recent studies have reported invasive fungal infections. For example, in a series of 120 consecutive AH patients admitted to an intensive care unit, 10% developed invasive fungal infections, including candidemia and invasive aspergillosis infections; all were exposed to corticosteroids ^81^.

Finally, early diagnosis of infection in AH is particularly challenging in patients with systemic inflammatory response syndrome (SIRS), which is diagnosed when two or more of the following criteria are met: (a) white blood cell count of more than 12,000/mL or less than 4,000/mL or more than 10% bands, (b) temperature of more than 38°C or less than 36°C, (c) heart rate of more than 90 beats/minute, (d) respiratory rate of more than 20/minute. In one study, SIRS was present in 20% of patients at admission and was associated with multiple organ failure and short-term death ^82^ independent of the presence of infection. Owing to the high rate of negative bacterial cultures in AH patients with SIRS, there is growing interest in the development of novel infection biomarkers in AH. Small studies have demonstrated efficacy of bacterial DNA and LPS ^79, 82^ to screen for infections, but further research is needed to validate these findings. As up to 26% of patients with AH initially present with infection ^80^, baseline screening for infection (blood cultures, urine cultures, chest radiography, and ascitic fluid analysis) is routinely performed; furthermore, a clinical trial studying the use of amoxicillin/clavulanic acid with prednisolone in patients with severe AH was nearing completion by December 2019 (ClinicalTrials.gov identifier: NCT02281929).

### Evaluation of nutritional status and nutritional support

Malnutrition—in particular, protein-calorie malnutrition—is a well-recognized complication of AH ^83, 84^. With the rising epidemic of obesity in Western nations, a subjective physical assessment by treating clinicians may incorrectly classify nutritional status in severe AH. Although a recent randomized clinical trial failed to demonstrate intense enteral nutrition via nasogastric tube superior to oral nutrition in patients with AH treated with corticosteroids, a subgroup analysis showed that patients receiving less than 21.5 kcal/kg per day had lower 6-month survival than patients meeting this threshold, and reduced survival was associated with decreased protein and lipid content in the diet ^85^. In addition, multiple macro- and micro-nutrient deficiencies have been described in AH. In particular, zinc deficiency is associated with increased intestinal permeability ^86^, and a clinical trial incorporating zinc supplementation (ClinicalTrials.gov identifier: NCT01809132) was completed in December 2018.

Recently, there has been renewed interest in the use of objective measures of nutritional status and physical function for outcome prediction in cirrhosis. In patients with decompensated cirrhosis, 6-minute walk test, cross-sectional abdominal muscle area, and gait speed are strongly associated with mortality, LT waitlist dropout, and increased hospital-related expenditures ^87– 89^. Furthermore, a liver frailty index composed of dominant grip strength, chair stands, and balance was recently shown to predict survival in decompensated cirrhosis ^90^. Although these tools have yet to be incorporated into prognostic models in AH, they may hold potential, in particular, for predicting long-term outcomes in short-term survivors of an episode of AH.

## Early liver transplantation for alcoholic hepatitis

Patients with AH who fail corticosteroids have poor long-term survival, and some studies reported up to 75% 6-month mortality ^44^. Therefore, LT has emerged as a potential curative therapy for AH. Historically, transplantation for alcoholic liver disease has required demonstration of abstinence for at least 6 months. The genesis of the “6-month rule”, however, was to permit recovery from an episode of acute-on-chronic liver failure after prolonged abstinence, but by the late 1990s, most US LT programs and insurance carriers required a 6-month abstinence period prior to approving LT listing. Multiple studies have examined the utility of the pre-transplant sobriety for predicting post-transplant relapse, but results have been conflicting. In one landmark analysis of LTs performed for alcoholic liver disease, steatohepatitis on explant pathology was used as a surrogate marker of recent alcohol consumption; interestingly, there were no differences in patient survival, graft survival, or alcohol relapse rates between subjects with and without steatohepatitis ^91^. Also, a systematic review of 22 studies reported that duration of pre-transplant abstinence was a poor predictor of post-transplant relapse ^92^, although some reports have identified duration of pre-transplant sobriety as a predictor of post-transplant drinking ^93– 95^. Interestingly, the United Network for Organ Sharing has never formally recommended a 6-month rule for transplant candidacy.

In a prospective, multi-center European trial, early LT for steroid non-responders afforded a marked survival benefit when compared with historical matched controls (6-month survival of 77% versus 23%, respectively; *P* <0.001) ^96^. Importantly, these transplants accounted for only 2.9% of all transplants performed at participating centers over the same period, thus allaying concerns regarding reduced donor availability for other liver diseases. Since this report, a number of single-center studies have reported similar survival benefit ^97, 98^. In particular, a recent US series of 147 LT recipients across 12 centers demonstrated a 3-year survival of 84%, and survival was 100% in patients who maintained sobriety after LT ^99^. Outcome modeling using data from the two seminal trials showed that early transplantation improves survival when compared with the 6-month probation—regardless of estimated post-transplant alcohol recidivism—and the highest survival advantage was among patients with a Lille score of 0.5 to 0.82 and a MELD score of at least 32 ^100^.

Reported psychosocial selection criteria across transplant centers are variable but appear to adhere to the following principles: (1) adequate psychosocial support, (2) a first episode of hepatic decompensation related to alcohol use, (3) absence of other substance use disorders, (4) absence of untreated psychiatric disease, and (5) acknowledgment of an AUD and willingness to participate in alcohol rehabilitation to maintain abstinence after LT. More recently, a model for alcohol relapse after LT identified prior alcohol-related legal issues and multiple failed alcohol rehabilitation attempts as additional risk factors ^99, 101^. Future studies are needed to optimize uniform selection criteria to (1) maximize long-term abstinence in LT recipients with severe AH and (2) ensure equity and fairness in the care of patients with severe AH.

## Conclusions

AH is a life-threatening form of acute-on-chronic liver failure in patients with chronic heavy alcohol use. Given temporal trends in alcohol consumption patterns worldwide, the incidence of AH, especially in young adults, is expected to rise in the next decade. Despite high rates of short-term mortality in severe AH, the treatment of AH and accompanying AUD has remained largely unchanged since the 1970s. A marked increase in the number of diagnostic and therapeutic trials in the last decade, however, reflects a growing interest in this devastating disease. LT is a life-saving therapeutic option for AH and is to be encouraged particularly in patients with a favorable profile and low risk for alcohol recidivism. Future studies should aim to improve post-transplant addiction support in order to maximize transplant benefits.
